# Efficient and rapid one-step method to generate gene deletions in *Streptococcus pyogenes*

**DOI:** 10.1128/spectrum.01185-24

**Published:** 2024-08-20

**Authors:** Lionel Schiavolin, Dalila Lakhloufi, Gwenaelle Botquin, Geoffrey Deneubourg, Corentin Bruyns, Jenny Steinmetz, Charlotte Henrot, Valérie Delforge, Pierre R. Smeesters, Anne Botteaux

**Affiliations:** 1 Molecular Bacteriology, European Plotkin Institute for Vaccinology (EPIV), Université libre de Bruxelles, Brussels, Belgium; 2 Department of Paediatrics, Brussels University Hospital, Academic Children Hospital Queen Fabiola, Université libre de Bruxelles, Brussels, Belgium; University of Florida College of Dentistry, Gainesville, Florida, USA

**Keywords:** *Streptococcus pyogenes*, knockout, rapid method

## Abstract

*Streptococcus pyogenes* or Group A *Streptococcus* (GAS) remains a significant infectious problem around the world, particularly in low- and middle-income settings. Moreover, a recent invasive GAS infection (iGAS) upsurge has been observed in high-income settings. However, to date, no vaccine is available. Finding a good vaccine antigen and understanding the role of virulence factors in GAS infections have been hampered, in part, by technical difficulties to transform the many different strains and generate knockout mutants. Using colE1-type plasmid as a suicide vector, we have set up a method allowing the generation of non-polar mutants of GAS in 3 days.

**IMPORTANCE:**

Group A Streptococcus (GAS) is a major human pathogen, causing diseases ranging from mild and superficial infections of the skin and pharyngeal epithelium to severe systemic and invasive diseases. Since June 2022, several European countries, the US, and Australia are facing an upsurge of invasive life-threatening GAS infections. Finding a good vaccine antigen and understanding the role of virulence factors in GAS infections have been hampered, in part, by technical difficulties to transform the many different GAS strains and generate knockout mutants. Moreover, these tools must be adapted to a large range of different strains, since GAS are divided into more than 260 emm-types (M-type). We have set up a method allowing the generation of non-polar mutants of GAS in 3 days and in diverse backgrounds, which contrasts with previously published protocols.

## INTRODUCTION

*Streptococcus pyogenes* or Group A *Streptococcus* (GAS) is responsible for a plethora of clinical manifestations, among which sore throat and impetigo are superficial and non-life-threatening, whereas necrotizing fasciitis or streptococcal toxic shock syndrome represent invasive and deadly infections (1). Overall, GAS infections are responsible for approximately 500,000 deaths/year in part due not only to invasive infections (160,000 deaths/year) but also to post-infection immune sequelae like rheumatic heart disease (2). Despite this high mortality rate, no licensed vaccine against GAS is commercially available.

If research on both vaccine antigen discovery and function in GAS pathogenesis has been hindered by several factors (3), one underestimated limiting factor is the lack of good genetic tools to efficiently manipulate GAS genomes. Moreover, these tools must be adapted to a large range of different strains, since GAS are divided into more than 260 *emm*-types. Their transformation and recombination abilities are different due to the thickness of the capsule, the presence of specific defense systems against foreign DNA such as CRISPR-Cas, Restriction-Modification (RM), or host-/prophage-encoded recombinases (4–6).

Over the past decades, efforts have been made to develop genetic tools to study gene function in GAS virulence and metabolism [reviewed in (7)]. One way to decipher the function of a gene is to generate, when possible, a knockout (KO) strain. This KO generation is generally done by double allelic exchange to limit the polar effect on downstream genes expression, organized in operon. The first step is to transform the bacteria with either a linear PCR product or a non-replicative or conditionally replicative plasmid (suicide vector). The second step relies on the homologous recombination between the host genome and the suicide vector that contains the flanking regions (FR) of the target gene, which is often replaced by an antibiotic resistance cassette. The recombination is based on host/prophage-encoded recombinases or could also be assisted by some recombinases present on a helper plasmid. In GAS, both steps, i.e., transformation and recombination (double allelic exchange), are difficult to perform. Indeed, GAS is a Gram-positive bacterium with a thick peptidoglycan layer, is encapsulated, and carries defense systems against foreign DNA, making it resistant to transformation. However, based on what has been done in *Enterococci* (8), electroporation of exponentially growing cells in the presence of glycine revolutionized transformation of GAS. Alternatively, use of sucrose has also be shown to improve GAS transformation efficiency (9). In highly encapsulated strains, addition of hyaluronidase has also been used (9).

Another hurdle is the presence of restriction-modification systems (RM) in all genotypes (4, 10, 11). However, solutions have been found, like knocking out RM encoding genes or adding the phage DNA mimic protein Ocr to the electroporation reaction (4, 10, 12).

Knockout strains of GAS have been generated using different methods, reviewed in (9, 13). Briefly, the use of non-replicative plasmids that are entirely integrated to disrupt the target gene by a single homologous recombination event is quick, easy and efficient but leads to a partial duplication of the target gene and has a strong polar effect on the expression of downstream genes. Linear DNA, which contains flanking regions of the target genes and an antibiotic resistance cassette (*aphA3*, *cat194,* or *ermC*), has been used to induce double allelic exchange in a single step. This technique has a very low success rate, which has generally been attributed to the rarity of double recombination in GAS, although this has not been proven experimentally. Moreover, polar effects have also been reported for this method, which could be due to the presence of transcription/translation termination signals in the antibiotic resistance cassette used (9).

To avoid this polar effect, protocols for in-frame deletion with or without the introduction of an antibiotic resistance cassette have been largely used (14–16). These methods rely on the use of thermosensitive vectors (pGhost5, pLZts, and pBFK) carrying the flanking regions of the target gene that have been either partially deleted by inside-out PCR (17), completely deleted by overlapping PCR (18) or replaced by an antibiotic resistance gene without any promoter/terminator region (9). These plasmids are then transformed into GAS at a permissive temperature for replication (30°C). Shifting to a non-permissive temperature (37°C) and maintaining the antibiotic pressure allows us to select clones with a single recombination event. At this step, the bacterial chromosome contains both the in-frame deletion and the wild-type allele. Shifting back to a permissive temperature allows the enrichment of bacteria in which the plasmid has been excised, due to conflicts between the replication origins of the chromosome and integrated plasmid. A third shift in temperature (37°C) without any selection allows the emergence of clones that have lost the plasmid, leaving either the deleted or wild-type allele. Allelic exchange is then confirmed by PCR and sensitivity to antibiotics on agar plates (9, 13, 18). Overall, the generation of such mutants is time consuming, ca. 7 days, and the workload is quite substantial (9, 13, 18). Moreover, temperature shifts must be well-controlled to avoid the generation of clones with a thermostable plasmid.

Counterselection methods to reduce the tough task of screening for loss of the suicide plasmid can be used to reduce the workload and rely on the addition of a conditionally toxic gene on the suicide plasmid (7). The *rpsL* WT allele, encoding a ribosomal protein, is the target of streptomycin and is dominant-negative over a mutated resistant allele (19). As GAS is not naturally resistant to streptomycin, one has to first generate a resistant strain by serial passage on increasing streptomycin concentration. Another strategy using a mutated *pheS* allele, encoding the Phenylalanyl-tRNA Synthetase, has been developed in *E. coli* (20) and successfully applied to *Streptococcus mutans* and *Enterococcus faecalis* (21). This mutant allele allows the misincorporation of 4-chloro-phenylalanine in proteins, which is toxic for bacteria and does not require modification of the target strain.

In this study, we describe a rapid method, i.e., 3 days (after suicide plasmid construction), to generate KO strains of GAS by double allelic exchange without any polar effect. We used a colE1-derived vector (pUC19), with a high copy number in *E. coli* and therefore high yields of pure DNA, which is non-replicative in GAS. The antibiotic resistance was adapted to GAS by replacing the *bla* gene (ampicillin) with *aad9* (spectinomycin) in the plasmid backbone and the target gene with the *aphA3* gene (kanamycin). The absence of thermosensitive replication allowed for a rapid selection of double recombinants. Finally, we also evaluated the efficacy of a mutated *pheS* gene (20) as a counter-selective marker for double recombination in GAS. The method was developed with a suicide plasmid that inactivates the *sagB* gene (part of the *sag* operon encoding a GAS hemolysin) in an M5 clinical isolate (22). The approach was then successfully applied to different genes and GAS backgrounds (M-types).

## MATERIALS AND METHODS

### Bacterial strains, culture media, and growth conditions

*E. coli* TOP10 strains (Invitrogen) were grown with shaking in Lysogeny broth (LB, Sigma) medium or statically on LB agar at 37°C. GAS strains were statically grown using agar or liquid Todd-Hewitt medium (Roth) supplemented with 0.5% yeast extract (Roth) (THY) at 37°C and 5% CO_2_. When needed, kanamycin (50 µg.mL^−1^ for *E. coli* and 300 µg.mL^−1^ for GAS), spectinomycin (100 µg.mL^−1^), or streptomycin (50 µg.mL^−1^) were added. GAS strains used in this study are the M5 LO1 (22), M75 emmy (23), M75 (24), M1T1 MGAS5005 (25), M25 NCTC8306 (ATCC 12204), and M98 (NS88.1) strains.

### Plasmid construction

All primers used in this study are listed in Table S1, and all plasmids are generated in Table 1. The *colE1* replication origin (1.181 bp) was amplified from the pUC19 vector using C2-colE1-F and D2’-colE1-R primers. The spectinomycin resistance gene (818 bp) was amplified from the pLZts using either B2-specR-F or I2-specR-F and C2’-specR-R primers. The promoter of the *bla gene* (ampicillin resistance) of the pUC19 was amplified using A2-pAmpi-F and B2’-pAmpi-R primers. The *pheS* gene (1.122 pb) was amplified using B2-pheS-F and I2’-pheS-R from a genomic DNA extract of the M25 strain. pheS** was generated by directed mutagenesis. The two substitutions T286S/A340G, corresponding to T251S/A294G mutations in *E. coli* PheS (20), were introduced into *pheS* to allow the misincorporation of the toxic 4-chloro-phenylalanine (20) for counterselection as suggested in (7, 20).

**TABLE 1:** Efficiency of mutant generation using the pKOSpy strategy in different genomic contexts and different M-types/strains^*a*^

Strains	Target gene	Number of reisolated transformants	Number of single recombinants	Number of double recombinants	% of success
M5 (LO1)	*sptR*	56	15	41	73
M75 (Emmy)	*mrp*	56	33	23	41
M75 (clinical strain)	*emm*	49	29	20	41
M25 (NCTC8306)	*emm*	64	57	7	11
M5 (LO1)	*isp2*	45	6	39	87
M1 (SF370)	*hasA*	30	2	28	93
M98 (clinical strain)	*enn*	NA	NA	NA	NA

^*a*^ NA : non-applicable.

Up- and down-stream flanking regions of *sagB*, approximately 1 kb in length, were amplified by PCR using D2-RF1*sagB*-F/Z2′-RF1*sagB*-R primers (997 bp) and E2-RF2*sagB*-F/A2′-RF2*sagB*-R (1033 bp) on a genomic DNA extract of the M5 LO1 strain (22). The *aphA3* gene (from ATG to TAA codons, 808 bp) was amplified from the pUC18K plasmid (26) using primers Z2-K7-F and E2′-K7-R. All seven fragments were digested with *Bsa*I (NEB) and ligated with T4 DNA ligase (NEB) by Golden Gate assembly as previously described (27) and transformed in TOP10 electrocompetent *E. coli*. Clones were selected on LB agar plate supplemented with spectinomycin.

For the other suicide plasmids, the vector (pKOSpy, 3.485 pb) was amplified from pKOSpy-*sagB* using D2’-colE1-R and A2-pAmpi-F primers. The pKOSpy, the *aphA3* gene, and the 1 kb flanking regions of the target genes were then subjected to Golden Gate assembly and *E. coli* transformation. All plasmids were checked by sequencing (Eurofins).

The pSWITCH was constructed by Golden Gate assembly (*Bsm*BI) using the pFD116 backbone (27) (A-repA-F and R’-Ter-R primers) and adding an inducible riboswitch (E) under the *sagA* promoter from pSIN-*murE* (28)(R-PsgR-F and Y’-PsagR-R primers), and two *Bsa*I sites (EmptyY-F and EmptyA-R primers) for further cloning. The *sagB* complementation plasmid (pSWITCH-*sagB*) was constructed by cloning the *sagB* gene into the pSWITCH plasmid using Y2’-sagB-F and A2’-sagB-R primers and confirmed by sequencing. *sagB* expression was induced by the addition of 2 mM -theophylline (VWR) to the medium. Both empty pSWITCH and pKOSpy-*sagB* plasmids are available from Addgene (222402 and 222345, respectively).

### Bacterial transformation and screening

The standard procedure was used to prepare electrocompetent TOP10 *E. coli* (29). Electrocompetent GAS strains were prepared as described previously, with slight modifications (30). Briefly, bacteria were grown to mid-log phase (OD_600_ of 0.3) and pelleted at 5,000 × *g* for 10 minutes at 4°C. The bacterial pellet was resuspended and washed twice with ice-cold 0.625M sucrose. In total, 100 mL of *E. coli* containing suicide plasmid was grown in Terrific Broth (Roth), followed by a plasmid Maxiprep (VWR, EZNA Plasmid MaxiKit). If needed, we then precipitated the plasmidic DNA according to the manufacturer’s instructions with slight changes (elution with H_2_O in 50 µL instead of in 200–500 µL of elution buffer). The suicide plasmid (50–100mg) or complementation plasmid (1 mg) (E.Z.N.A. Plasmid DNA Maxi Kit, Omega Bio-Tek) was added to 80 µL of bacteria (concentrated 250×, vol/vol from bacterial growth) and electroporated with a Micropulser electroporation system (Bio-rad) at 2.5 kV, 25 µF capacitance, and 200 Ω in a 0.2 cm cuvette. Bacteria were then quickly placed in 10 mL THY + 0.25M sucrose without antibiotics and allowed to recover for 2 h at 37°C. After recovery, transformed cells were centrifuged at 5,000 × *g* for 10 minutes at room temperature and resuspended in 300 µL of THY. In addition, 100 µL of bacteria were finally plated on THY agar supplemented with the appropriate antibiotic (kanamycin for KO generation and spectinomycin for complementation) and incubated overnight at 37°C with 5% CO_2_. For KO mutant generation, the resulting clones were then streaked on two THY agar plates containing either kanamycin or spectinomycin and grown overnight at 37°C to discriminate between single (Kan^R^ Spec^R^) and double (Kan^R^ Spec^S^) recombinants. Double recombinants were validated by PCR on genomic DNA prepared from colony with InstaGene Matrix (Bio-Rad) using GoTaq G2 DNA Polymerase (Promega) and primers listed in Table S1.

In case of bacterial lawn of transformants, bacteria were recovered in 5 mL of THY, grown 16 h at 37°C with 5% CO_2_ with kanamycin, diluted, and plated on 4 CP (Sigma)-containing plate (0.4%) to kill simple recombinant clones which contain pheS**.

### RT-qPCR

GAS strains grown in THY to exponential or stationary phase were harvested and resuspended in the RNA protect buffer (NEB). After bacterial lysis using a FastPrep 5G beads disruptor (MP-Biomedicals), RNA extractions were performed using the Monarch Total RNA Miniprep kit (NEB). Bacterial DNA was removed using TURBO DNA-free Kit (Thermofisher). cDNA synthesis was done using the Protoscript II First Strand cDNA Synthesis (NEB). Finally, qPCR was conducted on a CFX96 or a CFX384 touch system (Bio-Rad) using the Luna Universal qPCR Master Mix (NEB), which uses SYBR Green for detection, and gene-specific primers listed in Table S1. *tuf* gene was used to normalize the data. Fold change (FC) was calculated compared with expression in the WT background.

### Quantitative hemolysis test

Sheep blood was centrifuged at 2,000 × *g* for 10 min at 4°C. The upper layers (plasma and buffy coat) were carefully discarded, and PBS was added to reach the initial volume. Ten milliliters of bacterial culture (exponential phase) were centrifuged, and the supernatant was filtered (filter pore 0.22 µm). Sheep red blood cells (100 µL) were incubated with 100 µL of bacterial supernatant for 3 h at 37°C. After centrifugation at 2,000 × *g*, the OD of the supernatants was measured at 540 nm.

## RESULTS

### Construction of the suicide plasmid pKOSpy

**Fig 1:**
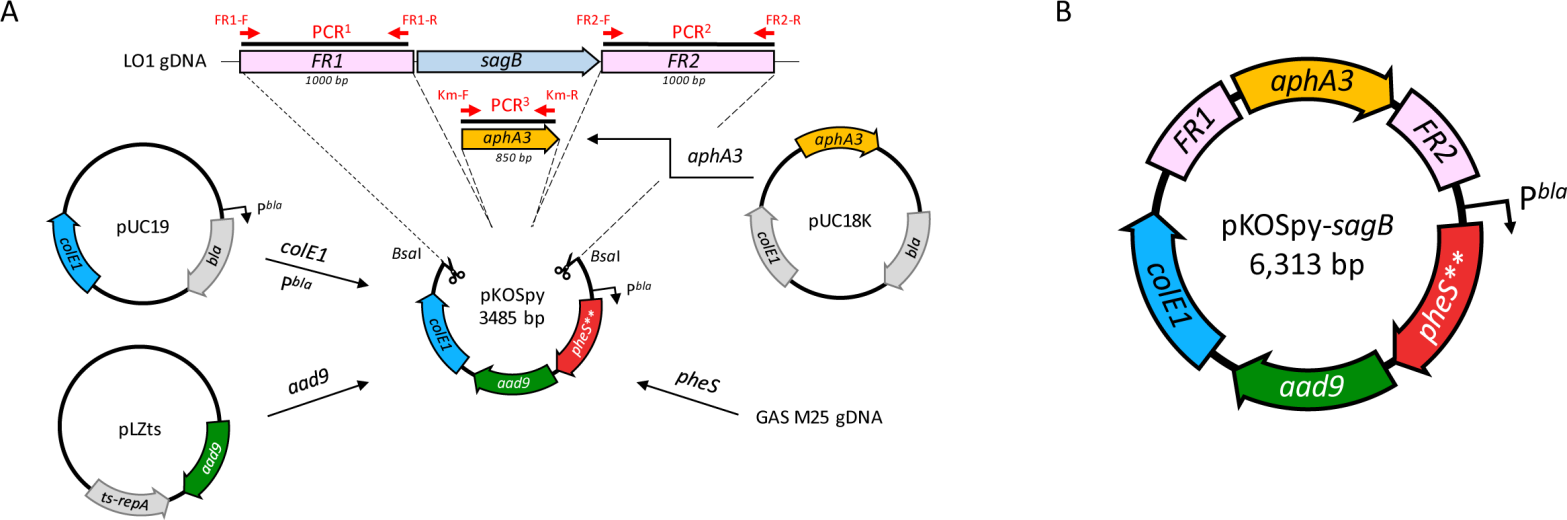
(**A**) Construction strategy for *sagB* allelic exchange with the pKOSpy vector. Upstream (FR1) and downstream (FR2) flanking regions of the *sagB* gene and the kanamycin resistance (33) gene was inserted using Golden gate assembly with *Bsa*I enzyme in the pKOSpy backbone consisting of the *colE1* ori and *bla* promoter (P*^bla^*) controlling the expression of the pheS** mutant allele and the *aad9* spectinomycin resistance gene. Primers used to amplify the three PCR fragments as well as origin of all fragments are depicted. (**B**) Map of the pKOSpy-*sagB* plasmid. Colored as in A. gDNA, GAS genomic DNA; bp, base pairs.

To construct a suicide plasmid for GAS, we decided to use the replication origin (ori site) of a pUC19 (colE1) (Fig. 1A), which cannot replicate in GAS but yields a high copy number of plasmids in *E. coli*. We chose to replace the ampicillin resistance gene with one encoding spectinomycin resistance since, for now, all GAS strains are still sensitive to b-lactams, which are the gold standard treatment for GAS infection. This resistance gene was cloned under the *bla* gene promoter allowing expression in both species. Finally, the modified *pheS* gene (pheS**) was added as a counter-selection option to help isolate double recombinants in poorly recombinogenic strains or genomic regions, e.g., lack of Chi sites (31), high density of DNA-binding proteins (32).

We first choose to mutate the *sagB* gene, encoding a 36 k-Da cytoplasmic protein essential for Streptolysin S (SLS) production, because of its easily detectable phenotype on blood agar plates. Indeed, SLS is one of the hemolysins of GAS allowing lysis of red blood cells, which can be observed on blood agar plates or monitored by measuring the optical density of released hemoglobin. Up- and down-stream flanking regions of *sagB* as well as a resistance gene to kanamycin (*aphA3*), were used to generate the suicide plasmid, named pKOSpy-*sagB* (Fig. 1B).

### Generation of sagB KO in M5

**Fig 2:**
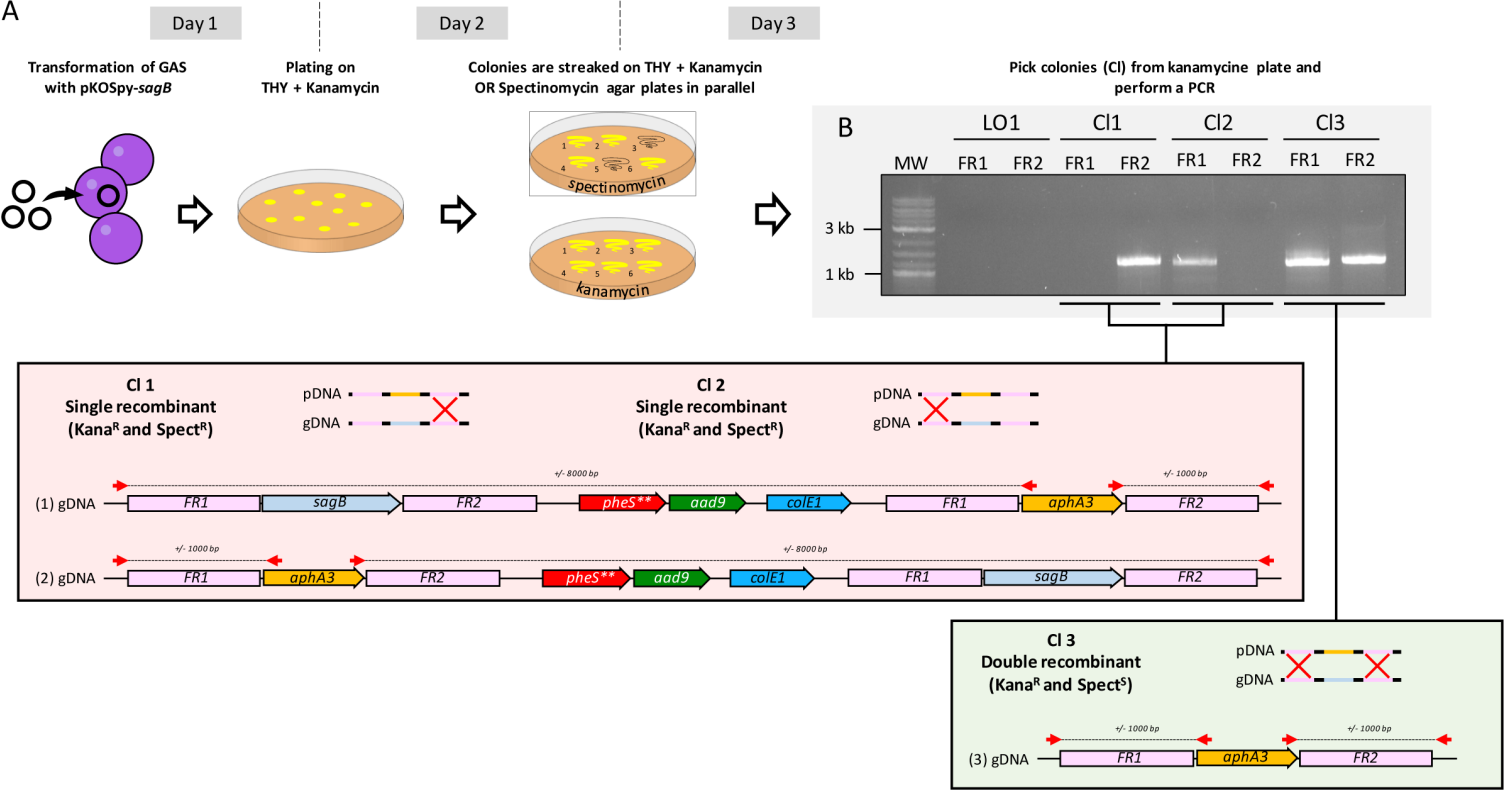
(**A**) Strategy used to generate GAS KO mutants in 3 working days. Day 1: the suicide vector is transformed in GAS cells and GAS cells are plated on THY supplemented with Kanamycin. Day 2: Kanamycin-resistant colonies are streaked on plates containing either kanamycin or spectinomycin. Day 3: Spectinomycin-sensitive and kanamycin-resistant colonies are screened by PCR using primers annealing outside the cloned FR1 and FR2 and in the *aphA3* gene. Double recombinants (Kanamycin^R^ and Spectinomycin^S^) present two bands at around 1,000 bp. Simple recombinants (Kanamycin^R^ and Spectinomycin^R^) present only one PCR band (either from FR1 or FR2). Red arrows indicate primer annealing. gDNA, genomic DNA; pDNA, plasmidic DNA; bp, base pairs. (**B**) PCR screening of the *sagB* mutant candidates by PCR. Clones 1 and 2 are spectinomycin^R^ and kanamycin^R^ (single recombinants, taken as an example), and clone 3 is spectinomycin^S^ and kanamycin^R^ (double recombinant). LO1, the wild-type strain; FR, flanking region.

The cloning and mutant generation strategies are depicted in Fig. 2A. After GAS M5 transformation, bacteria were selected on plates containing kanamycin. After streaking eight clones on kanamycin or spectinomycin, we performed PCR with primers annealing outside the flanking regions and inside the kanamycin gene to check the correct localization of the recombination events. All selected clones with a spectinomycin-sensitive (spect^S^) and kanamycin-resistant (kana^R^) profile exhibited two PCR products at the expected size (Fig. 2B). The WT M5 strain and two clones with a spectinomycin- and kanamycin-resistant profilewere used as control. No PCR product was detected in the WT strain, whereas only one PCR product was detected in the single recombinant clones (Fig. 2B) since the second flanking region, containing the integrated plasmid, cannot be amplified (around 8,000 bp).

**Fig 3:**
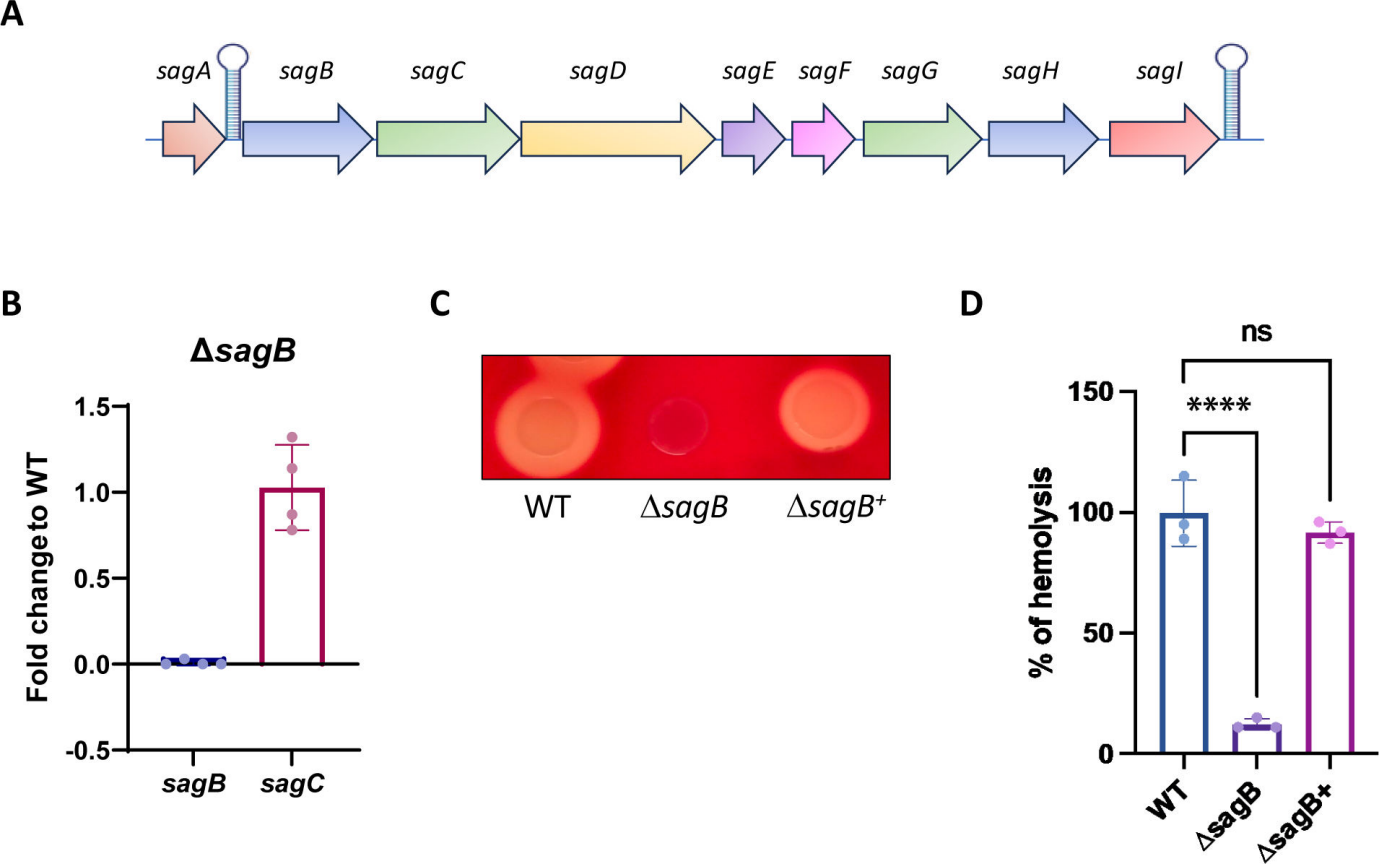
(**A**) Schematic representation of the *sag* operon responsible for Streptolysin S production in GAS. A rho-independent terminator sequence is schematized between the *sagA* and *sagB* gene and at the end of the *sag* operon. (**B**) Quantification by RT-qPCR of the fold changes of *sagB and sagC* RNA transcripts between the *sagB* mutant (*DsagB*) compared with the WT strain. (**C**) Phenotypic validation of the *sagB* mutant (D*sagB*) and its complemented derivative (D*sagB^+^*) compared with the parent LO1 strain (WT) on 5% sheep blood agar plates. Bacteria with functional hemolysin show a clear halo around the colonies. (**D**) Quantitative analysis of SLS activity of the *sagB* mutant (D*sagB*) and its complemented derivative (D*sagB^+^*) compared with the parent LO1 strain (WT). Release of hemoglobin was measured after incubation of bacterial supernatant with red blood cells for 3 h at 37°C. The percentage of hemolysis has been calculated compared with the WT strain, considered as 100%. A one-way ANOVA test was used for statistical analysis.

The absence of a polar effect by the *aphA3* gene on the downstream gene was then checked by RT-qPCR. The *sag* operon consists of nine genes (A to I) involved in the synthesis and secretion of hemolysin, known as SLS (Fig. 3A) (34). Since *sagB* is located just before *sagC* in the *sag* operon (Fig. 3A), we monitored the expression of *sagC* in the *sagB* mutant (Δ*sagB*) and compared it with the expression level in the wild-type strain. As shown in Fig. 3B, no change in *sagC* expression was observed, confirming the absence of polar effect.

### Phenotypic characterization of the sagB mutant

As the product of *sagB* is responsible for the maturation of SLS (encoded by *sagA*), we next plated the WT and the *sagB* mutant (*DsagB*) on 5% sheep blood agar plates. A complementation plasmid, named pSWITCH, was constructed by cloning the *sagB* gene under the control of a riboswitch (35) (Fig. S1) and transformed into the *DsagB* strain (*DsagB^+^*). As seen in Fig. 3C, the mutant exhibits the expected phenotype (36) and seems well complemented by the introduction of the *sagB* gene, in contrast to previous attempts of complementation (36). We then performed a quantitative hemolysis assay and showed that the hemolytic activity of the complemented strain was similar to the WT strain in contrast to the *DsagB* strain (Fig. 3D).

### Mutant generation efficiency in different M-types and counter-selection method

To test the efficacy of our method on different M-types and different genomic contexts, we generated several KO strains, listed in Table 1. All suicide plasmids were constructed using the pKO-Spy backbone (PCR using D2’-colE1-R and A2-pAmpi-F primers), the *aphA3* gene, and 1 kb up- and down-stream flanking regions of the target genes (Table S1). Mutants of *emm25*, *emm75*, *mrp24*, *enn314*, *isp2*, and *sptR* genes were generated in different GAS backgrounds, i.e., M5, M25, M75, and M98 strains, with different success rates. The same protocol used for generating the *sagB* knockout was applied to all *emm* types. All mutants were checked by PCR, and the absence of polar effect was checked by RT-qPCR (Fig. S2).

For the *enn314* KO generation in M98, we obtained a bacterial lawn after transformation. We therefore used the counter-selection with 4 CP to discriminate between single and double recombinants (Fig. S3). Bacteria were scrapped and allowed to grow in TSB. Serial dilutions were plated on THY- 0.4% 4 CP agar, and isolated clones were checked by PCR (Fig. S3A and B). All clones growing in the presence of 4 CP were double recombinants, and we confirmed that single recombinants were not able to grow on 4 CP (Fig. S3C).

## DISCUSSION

The present study revisits the use of colE1-type suicide vector to generate mutants in GAS by double allelic exchange without polar effects. It offers remarkable advantages in terms of mutant generation speed compared with conventional techniques (9). Indeed, this method eliminates the need for multiple passages and temperature shifts, reducing the time required to generate mutants from 13 days to just 3 days.

The pKOSpy is composed of the high-copy colE1 origin of replication, allowing an increased plasmid yieHIld in *E. coli,* which is required to generate mutant with non-Creplicative plasmid in GAS (up to 100 µg of plasmids is sometimes required). It carries the counterselection PheS** variant that incorporates the toxic 4-chlorophenylalanine (20) into proteins. This allows the counterselection of single recombinants if the targeted DNA regions or the selected GAS strain are less recombinogenic or if an in-frame deletion without antibiotic resistance gene is considered. Although we did not face such issues, we used 4 CP counterselection and successfully isolated double recombinants only for the *enn314* (M98) KO strain.

DNA manipulation of bacteria could impact expression of downstream genes (polar effect of the antibiotic resistance gene) or cause spurious mutation in the genome, which can both account for the phenotype. The mutant needs to be validated by checking gene expressions and performing a complementation assay. To validate the non-polar effect of the kanamycin resistance genes on the expression of the downstream genes, we determined their expression by RT-qPCR. We did not observe significant differential expression between the WT and deletion mutant strains, in contrast to expression of the deleted gene. Moreover, as a proof of concept, we generated a teophylline-inducible plasmid (pSWITCH) expressing *sagB* since the previous attempt to complement the *sagB* mutant was partially successful (36). The pSWITCH-*sagB* was found to fully complement the *sagB* mutant phenotype (i.e., red blood cell lysis).

Besides speed advantages, our approach has some limitations. First, it relies on a non-replicative plasmid that requires huge DNA quantities for GAS transformation. Indeed, the *hsd* restriction-modification system can reduce transformation efficiency (4) and most likely the formation of recombinants by targeting the suicide plasmid. To increase the success of this method, strategies to improve the efficiency of transformation and therefore recombination in GAS would be ideal. The use of inhibitors of type I restriction systems, like Ocr, would likely decrease the amount of plasmid DNA required (12). Second, the replacement of the target gene CDS with that of an antibiotic resistance gene requires sufficient expression from the endogenous promoter to select the KO mutants. In addition, the use of an antibiotic resistance marker constrains the potential to generate multiple knockout in the same background. These concerns warrant further research to improve the present method, notably by means of 4 CP counterselection to generate untagged deletion mutant. Recently, CRISPR Cas9-based techniques have been efficiently used in Gram-positive bacteria such as *Streptococcus pneumoniae*, *Staphylococcus aureus,* and *Streptococcus equisimilis* (37–39). As *cas9* originates from GAS, its use to generate in-frame deletion should be easier, with the transformation of a plasmid expressing a targeting sgRNA in Cas9 expressing strains. However, no Cas9-based techniques in GAS have been published yet. An interference system using dCas9 could be useful to transiently shut down the expression of genes. However, the ectopic and transient expression of Cas9 in GAS could affect the expression of an array of GAS genes as Cas9 is described as a global regulator of GAS virulence and physiology (40).

In conclusion, the proposed m simplifies and accelerates the process of mutant generation in diverse backgrounds, including clinical strains. Additionally, we validated the use of 4 CP as a counterselection method for GAS and brought an additional tool to express genes in a controlled manner *in trans*.

## SUPPLEMENTAL MATERIAL

Supplemental figures spectrum.01185-24-s0001.pdf Fig. S1 to S3.

Supplemental material spectrum.01185-24-s0002.docx Captions for Fig. S1 to S3.

Table S1 spectrum.01185-24-s0003.docx Primers and plasmids used.

ASM does not own the copyrights to Supplemental Material that may be linked to, or accessed through, an article. The authors have granted ASM a non-exclusive, world-wide license to publish the Supplemental Material files. Please contact the corresponding author directly for reuse.
