# Supplementary figures and images for: Efficient and rapid one-step method to generate gene deletions in Streptococcus pyogenes
Source: Microbiol Spectr. 2024 Aug 20;12(10):e01185-24. doi: 10.1128/spectrum.01185-24 (PMC11448258; doi:10.1128/spectrum.01185-24)

Fig.S1

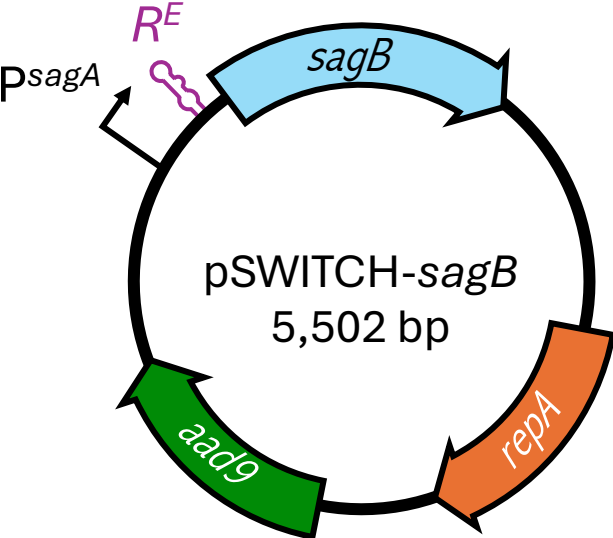

Fig.S2

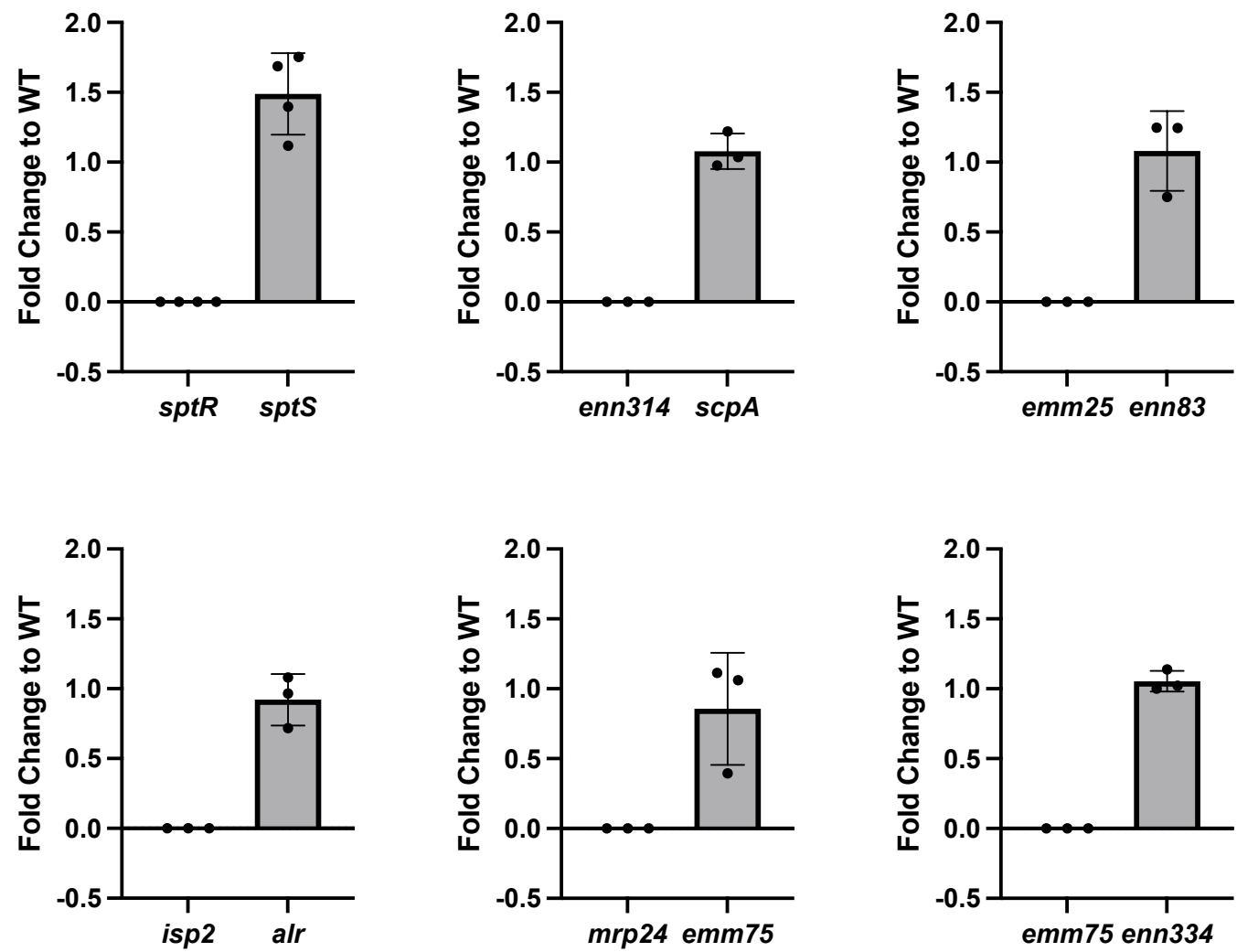

**Fig.S3**

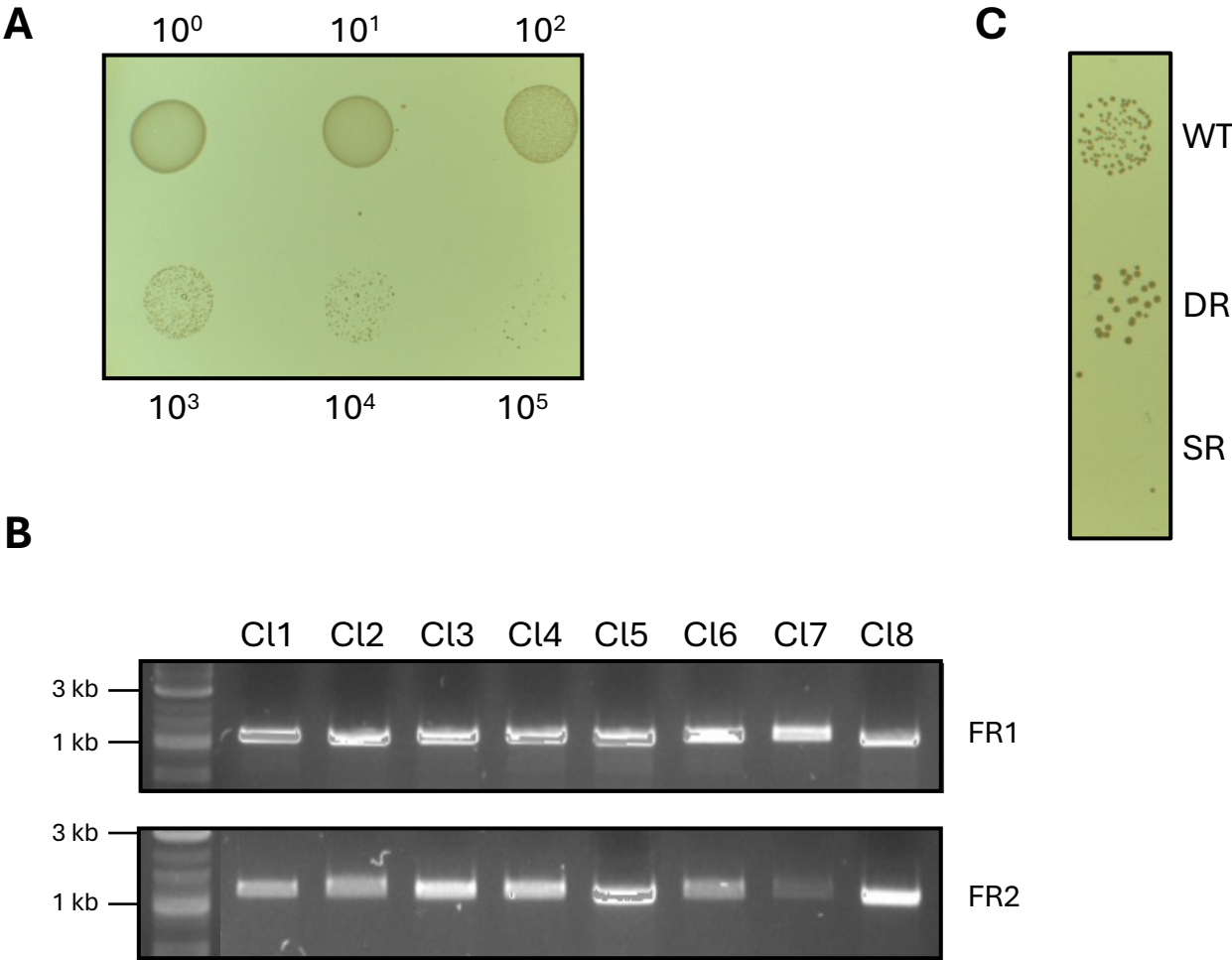

Supplement: Supplemental figures — Fig. S1 to S3. [file spectrum.01185-24-s0001.pdf]
