# Supplementary material for: Efficient and rapid one-step method to generate gene deletions in Streptococcus pyogenes
Source: Microbiol Spectr. 2024 Aug 20;12(10):e01185-24. doi: 10.1128/spectrum.01185-24 (PMC11448258; doi:10.1128/spectrum.01185-24)
Supplement: Supplemental material — Captions for Fig. S1 to S3. [file spectrum.01185-24-s0002.docx]

**Supp. Figures caption**

**Fig.S1**: Map of the pSWITCH-*sagB* plasmid. The *sagB* gene from the LO1 strain has been cloned under the *sagA* promoter (P^sagA^) from MGAS315 and the riboswitch E (R^E^) from pSIN-*murE* plasmid. The riboswitch represses translation in absence of theophillin. The *aad9* gene, allowing spectinomycin resistance, and the origin of replication were cloned from the pFD116.

**Fig.S2**: Expression of the knocked-out and the downstream genes in the different GAS mutants was determined by RT-qPCR and plotted as a fold change (FC) relative to their parent strains (WT). *tuf* was used as a housekeeping gene to normalize the data.

**Fig.S3**: **A/** Isolation of *enn314* clones onto THY agar supplemented with 0.4% 4CP. Transformants were first resuspended in THY, grown overnight with kanamycin, diluted and plated on THY agar + 0.4% 4CP. **B/** PCR screening of eight of the isolated *enn314* mutant candidates (Clones, Cl). Primers hybridize outside the flanking regions (FR1 or FR2) and inside the *aphA3* gene as in Figure 2. **C/** LO1 (WT), double (DR) et single (SR) recombinants of *enn314* mutant on THY agar + 0.4% 4CP.
