# Supplementary material for: Efficient and rapid one-step method to generate gene deletions in Streptococcus pyogenes
Source: Microbiol Spectr. 2024 Aug 20;12(10):e01185-24. doi: 10.1128/spectrum.01185-24 (PMC11448258; doi:10.1128/spectrum.01185-24)
Supplement: Table S1 — Primers and plasmids used. [file spectrum.01185-24-s0003.docx]

**Supp. data**

Table S1. Primers and plasmids used in this study

| Name | Sequence | Target | Source |
| --- | --- | --- | --- |
| Z2-K7-F | AGGTCTCTCATTATGGCTAAAATGAGAATATCACCGGA | *aphA3* | pUC18K |
| E2’-K7-R | AGGTCTCTCGTCCTAAAACAATTCATCCAGTAAAATATAATATTTTATTTTC | *aphA3* | pUC18K |
| C2-ColE1-F | AGGTCTCTTTGGCTGTCAGACCAAGTTTACTCATATATAC | *colE1* | pUC19 |
| D2’-ColE1-R | AGGTCTCTATGTAGCTGTTTCCTGTGTGAAATTGTTATC | *colE1* | pUC19 |
| B2-specR-F | AGGTCTCTTTACGTGAGGAGGATATATTTGAATACATA | *aad9* | pLZts |
| C2’-specR-R | AGGTCTCTCCAAGTGTTTCCACCATTTTTTCAATTTTTTTA | *aad9* | pLZts |
| A2-pAmpi-F | AGGTCTCTATTAGCCAGCCCCGACACCC | *bla* promoter | pUC19 |
| B2’-pAmpi-R | AGGTCTCTGTAAACTCTTCCTTTTTCAATATTATTGAAGCATTTAT | *bla* promoter | pUC19 |
| B2-pheS-F | AGGTCTCTTTACATGGTTCCTCGTGTCAACGCT | *pheS* | NCTC8306 |
| I2’-pheS-R | AGGTCTCTCTTTACAGAGGTCTTCAAACGACTCG | *pheS* | NCTC8306 |
| I2-specR-F | AGGTCTCTAAAGGAAGAGTTTACGTGAGGAGGATATATTTGAATACATA | *aad9* | pLZts |
| D2-RF1sagB-F | AGGTCTCTACATCGTGGTATAGGTTTGTCCAGGTA | RF1 *sagB* | LO1 |
| Z2’-RF1sagB-R | AGGTCTCCAATGTAATTATTATTAGCTTTATAGTTCATCAAAAAATGTTGAC | RF1 *sagB* | LO1 |
| E2-RF2sagB-F | AGGTCTCTGACGTCTCAATGAAATATCAACTTAATAGTAATGTTCG | RF2 *sagB* | LO1 |
| A2’-RF2sagB-R | AGGTCTCTTAATCGTGTTGATATCTGCTGATCCTCATA | RF2 *sagB* | LO1 |
| endaphA3-F | CAAGCCTGATTGGGAGAAAAT | 3’-end aphA3 | N.A. |
| aphA3-R | TTCCTTCCGTATCTTTTACGC | 5-end aphA3 | N.A. |
| checkKOsagB-F | CTAGTGGACTCATTTATCCTGAGC | *eno* | N.A. |
| checkKOsagB-R | CATCGTGAGGATGGTTGC | *sagD* | N.A. |
| D2-RF1emm25-F | AGGTCTCTACATAGGTTATGTGCAAACCATCCAAAAC | RF1 *emm* | NCTC8306 |
| Z2'-RF1emm25-R | AGGTCTCCAATGTATTTGCTCCTTATTTTTTCATCTTTAGGGAA | RF1 *emm* | NCTC8306 |
| E2-RF2emm25-F | AGGTCTCTGACGAATATAAAAGGGATCAATGATGATTAACAAATTTC | RF2 *emm* | NCTC8306 |
| A2'-RF2emm25-R | AGGTCTCTTAATAGCATCATATTTAAATGATTCTACGCTTTTAG | RF2 *emm* | NCTC8306 |
| check-emm25-F | CTTAAAAACCAACAAGCCCTTGAAGC | *mrp* | N.A. |
| check-emm25-R | ATTTTGCATTTACATTTCTAAGGTCAGCA | *enn* | N.A. |
| D2-RF1sptR-F | AGGTCTCTACATTGCACATCAACTTGATCTGCTTGAA | RF1 *sptR* | LO1 |
| Z2'-RF1sptR-R | AGGTCTCCAATGGGTTACTCTTCCTCCGTCGTTT | RF1 *sptR* | LO1 |
| E2-RF2sptR-F | AGGTCTCTGACGGAGGTGACATGTTTAATCGTATCC | RF1 *sptR* | LO1 |
| A2'-RF2sptR-R | AGGTCTCTTAATAACTTCTAATTTCGCTCGTTTTCGTC | RF1 *sptR* | LO1 |
| check-sptR-F | TTTTACCACTTGGTGTGTAAGCTGTTC | *lamri00806* | N.A. |
| check-sptR-R | CCTTTTTCTTTGCTATTATGGGATTCGTCT | *sptS* | N.A. |
| D2-RF1enn314-F | AGGTCTCTACATAGCAGAGCGAAAAGAAAAAGAAGCA | RF1 *enn* | M98 |
| Z2'-RF1enn314-R | AGGTCTCCAATGTATCTACTCCTTAATTTTATGAAAAAGAAAATTAATAGT | RF1 *enn* | M98 |
| E2-RF2enn314-F | AGGTCTCTGACGGCCTTTAGAACTTGGTTTTTGTAACG | RF2 *enn* | M98 |
| A2'-RF2enn314-R | AGGTCTCTTAATTCTAGGCGGTAAGGTTCTTTCG | RF2 *enn* | M98 |
| CDC1 | TATTCGCTTAGAAAATTAA | *emm* | N.A. |
| check-enn314-R | CGAGCATAGTCTGCTAGTCC | *scpA* | N.A. |
| D2-RF1mrpy-F | AGGTCTCTACATGTATACCATAGCTGAAATGT | RF1 *mrp* | Emmy |
| Z2'-RF1mrpy-R | AGGTCTCCAATGTGTTTACTCCTTATTTCTTA | RF1 *mrp* | Emmy |
| E2-RF2mrpy-F | GGGTCTCTGACGGCCTAACCCACACTATCTTTTCTAG | RF2 *mrp* | Emmy |
| A2'-RF2mrpy-R | AGGTCTCTTAATCTTGAAGTTTGCTATTTGCTTCTGC | RF2 *mrp* | Emmy |
| check-mrpKO-F | ACAAGTCAACAGTGGAGAGAACT | *mga* | N.A. |
| check-mrpKO-R | CTTGCTTGTAACTCAGCTTTTTCTT | *emm* | N.A. |
| D2-RF1emm75-F | AGGTCTCTACATAGCCATTGAAGGTTATGTGC | RF1 *emm* | Clinical isolate M75 |
| Z2'-RF1emm75-R | AGGTCTCCAATGTATTTGCTCCTTATTTTTTC | RF1 *emm* | Clinical isolate M75 |
| E2-RF2emm75-F | GGGTCTCTGACGGCTATTAGACTGATGCTAAAGCTAAGAG | RF2 *emm* | Clinical isolate M75 |
| A2'-RF2emm75-R | CGGTCTCATAATTGATAATTTCTTACTTTCTTCAAGCGC | RF2 *emm* | Clinical isolate M75 |
| check-emm75-F | AGCTCAATCTCAGCATCACCAA | *mga* | N.A. |
| check-emm75-R | AGGGCTTTTCCTTGTGCATCTA | *enn* | N.A. |
| D2-RF1isp2-F | TGGTCTCTACATCGTCAAGCAGGTATTGATAAAGAG | RF1 isp2 | LO1 |
| Z2’-RF1isp2-R | TGGTCTCCAATGTAATTAGTCACTTCTTTCTAATGTCC | RF1 isp2 | LO1 |
| E2-RF2isp2-F | AGGTCTCTGACGTAACTAATGACTTTTCTTAAGGCTTAATT | RF2 isp2 | LO1 |
| A2’-RF2isp2-R | AGGTCTCTTAATACCTTTGTGATTTGCTGAGTCAA | RF2 isp2 | LO1 |
| check-isp2-F | TCAAGTGGATGGGTACTGTGT | *iniA* | N.A. |
| check-isp2-R | GCC CTG TCA TAT TCG AGA CC | *alr* | N.A. |
| EmptyY-F | CAAGTGAGACCGCAAGTGGTCTCG | N.A. | N.A. |
| EmptyA-R | CGAGACCACTTGCGGTCTCATAAT | N.A. | N.A. |
| Y2’-SagB-F | AGGTCTCTACATTTTAGCATCTCTATGTGATAGTGATATTAAG | *sagB* | LO1 |
| A2’-SagB-R | AGGTCTCTTAATTCATTGAGACTCCTTAGTTCCTACG | *sagB* | LO1 |
| R-pSagR-F | ACGTCTCTCTTAACGGTAAGACAGAAATCGGTCATTC | pSagA+ Riboswitch | pSIN |
| Y’-pSagR-R | ACGTCTCACTTGTTGTTACCTCCTTAGCAGG | pSagA+ Riboswitch | pSIN |
| R-ter-R | ACGTCTCTTAAGAGGTGAGCCAGTGTGACTCTAG | backbone | pFD116 |
| A-repA-F | ACGTCTCTATTATGGAGATCTGTCCATACCCATG | backbone | pFD116 |
| qPCR-sagB-F | TGCTTCAGGTGGAGGTTTATACC | *sagB* | N.A. |
| qPCR-sagB-F | GCATTAATAGCACCGTATTCCGC | *sagB* | N.A. |
| qPCR-sagC-F | GATGTGAGCCAGGCCTATTTACC | *sagC* | N.A. |
| qPCR-sagC-R | CCTGAATCTCAAGCGTCGGTAAG | *sagC* | N.A. |
| qPCR-tuf-F | AACTACTTTAACAGCTGCAATCACAACT | *tufA* | N.A. |
| qPCR-tuf-R | AGAAGCGTAATCTTTTGGTTGGTT | *tufA* | N.A. |
| qPCR-emmX-F | GCAAATAGCAAACTTCAAGCCC | *emmX* | N.A. |
| qPCR-emmX-R | CTTGCTTGTAACTCAGCTTTTTCTT | *emmX* | N.A. |
| qPCR-enn-F | AGGGCTTTTCCTTGTGCATCTA | *enn* | N.A. |
| qPCR-enn-R | TCAACTCTCAGCGCTTGAAGAA | *enn* | N.A. |
| qPCR-scpA-F | CCCGGCCAAGATATTTTGTCATC | *scpA* | N.A. |
| qPCR-scpA-R | AGACGCTCTGATGGTGTCATATC | *scpA* | N.A. |
| qPCR-sptR-F | CATGATGCCGATTAAGTCTGGC | *sptR* | N.A. |
| qPCR-sptR-R | AATCATCAGCTCCCGCATCTA | *sptR* | N.A. |
| qPCR-sptS-F | CTAAGTCTGGGGAAGAAAGAGGC | *sptS* | N.A. |
| qPCR-sptS-R | CCTCCAAAGGCCAACATCATTG | *sptS* | N.A. |
| qPCR-mrp-F | TGACTGACTTGCAAGCTAAGCT | *mrp24* | N.A. |
| qPCR-mrp-R | TTCACGCTTAGCTGAACCTAGG | *mrp24* | N.A. |
| qPCR-isp2-F | CGGTTTTGGCTGGTCAGTTG | *isp2* | N.A. |
| qPCR-isp2-R | GGTTCTTCGGACAACAAGGC | *isp2* | N.A. |
| qPCR-alr-F | GCTTTTGACACCTGGACAGC | *alr* | N.A. |
| qPCR-alr-R | AGTTTCCATCGCCCAACAGT | *alr* | N.A. |
| **Name** | **Description** | **Resistance** | **Source** |
| pSIN-MurE | Suicide plamside to generate murE inducible mutant | spectino | (28) |
| pFD116 | Ptet d*cas9* term PpflB sgRNA (BsaI) oriT in pLZ12 | spectino | (27) |
| pLZts | Thermosensitive vector derivative of pLZ12 (*aad9*) | spectino | (13) |
| pUC18K | *colE1* vector | kanamycin | (26) |
| pUC19 | colE1 vector | ampicillin | Addgene #50005 |
| pSWITCH | Empty inducible vector for complementation in GAS  Psag RBSE repA | spectino | This study |
| pSWTCH-sagB | pSwicth containing the *sagB* gene | spectino | This study |
| pKOSpy | Empty suicid vector for GAS | spectino | This study |
| pKO-sagB | pKO-spy containing the up and downstream flanking region of the *sagB* gene | spectino | This study |
| pKO-mrp24 | pKO-spy containing the up and downstream flanking region of the *mrp24* gene | spectino | This study |
| pKO-emm75 | pKO-spy containing the up and downstream flanking region of the *emm75* gene | spectino | This study |
| pKO-sptR | pKO-spy containing the up and downstream flanking region of the *sptR* gene | spectino | This study |
| pKO-enn314 | pKO-spy containing the up and downstream flanking region of the *enn314* gene | spectino | This study |
| pKO-emm25 | pKO-spy containing the up and downstream flanking region of the *emm25*  gene | spectino | This study |
| pKO-isp2 | pKO-spy containing the up and downstream flanking region of the *isp2* gene | spectino | This study |
